# Identification by Liquid Chromatography–Tandem Mass Spectrometry and Liquid Chromatography–Quadrupole Time-of-Flight Mass Spectrometry of the Contributor to the Thyroid Hormone Receptor Agonist Activity in Effluents from Sewage Treatment Plants

**DOI:** 10.1021/acs.est.2c02648

**Published:** 2022-09-13

**Authors:** Ryo Omagari, Mayuko Yagishita, Fujio Shiraishi, Shoji F. Nakayama, Masanori Terasaki, Tetsuya Tanigawa, Ichiro Yamauchi, Takuya Kubo, Daisuke Nakajima

**Affiliations:** † Health and Environmental Risk Division, National Institute for Environmental Studies (NIES), Tsukuba City, Ibaraki 305-8506, Japan; ‡ Department of Life and Environmental Science, Prefectural University of Hiroshima, Shobara City, Hiroshima 727-0023, Japan; § Graduate School of Arts and Sciences, Iwate University, Morioka City, Iwate 020-8550, Japan; ∥ Graduate School of Engineering, Kyoto University, Katsura, Nishikyo-ku,Kyoto 615-8510, Japan; ⊥ Department of Diabetes, Endocrinology and Nutrition, Kyoto University Graduate School of Medicine, Kyoto 606-8507, Japan; # Department of Material Chemistry, Graduate School of Engineering, Kyoto University, Katsura, Nishikyo-ku, Kyoto 615-8510, Japan; ∇ Graduate School of Pharmaceutical Sciences, Chiba University, Chiba City, Chiba 260-8675, Japan

**Keywords:** LC−MS/MS, LC−QToF−MS, sewage treatment plant, human thyroid hormone receptor, TRIAC

## Abstract

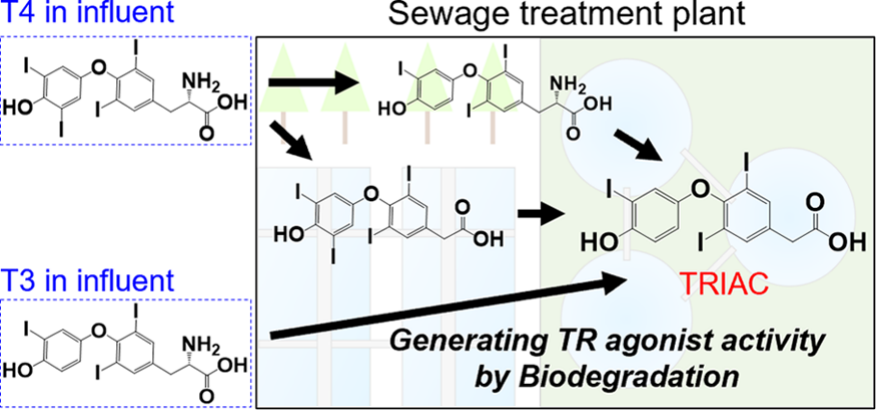

3,3′,5-Triiodothyroacetic acid (TRIAC) was identified as a major contributor to the activity of thyroid hormone receptor (TR) agonists in environmental water. TRIAC contributed 60–148% of the TR-agonist activity in effluents from sewage treatment plants (STPs). Meanwhile, the contributions of 3,5,3′-triiodothyronine (T3), 3,3′,5,5′-tetraiodothyronine (T4), and analogues were <1%. TRIAC concentrations in the range of 0.30–4.2 ng/L are likely enough to cause disruption of the thyroid system in living aquatic organisms. The origin of TRIAC in the STP effluents was investigated by analyzing both STP influents and effluents. Relatively high concentrations of T3 and T4 (2.5 and 6.3 ng/L, respectively) were found only in the influents. TRIAC was identified only in the effluents. These findings suggested that T3 and T4 in STP influents were potentially converted into TRIAC during activated sludge treatment or by other means. The evaluation of TRIAC at relevant environmental concentrations by *in vivo* assays and an appropriate treatment to reduce the TR activity in sewage are needed.

## Abstract 2

The contributor to the thyroid hormone receptor agonist activity in environmental water was unknown. This study reports that the contributor is TRIAC.

## Introduction

The thyroid hormones (THs) 3,5,3′-triiodothyronine (T3) and 3,3′,5,5′-tetraiodothyronine (T4) play significant roles in essential processes in humans and wildlife, such as their development, metabolism, and homeostasis.^1^ Therefore, if chemical compounds disrupt their system, adverse effects including tachycardia, atrial arrhythmias, and heart failure arise.

Some studies have reported developmental toxicity by xenogenous T3, T4, and TH analogues. Pigment loss on the skin and formation of blue–green pigments in the liver were observed in zebrafish (*Danio rerio*) exposed to 3,3′,5-triiodothyroacetic acid (TRIAC); an association of those phenotypes to reductions in the proliferation and survival of melanophores, as well as accumulation of biliverdin as an intermediate of heme catabolism, was suggested.^2^ Other studies reported similar outcomes.^3,4^ Malformations of zebrafish exposed to T3, T4, and TRIAC were also shown in those reports. Moreover, notable upregulation of TH signaling genes, such as TH receptors (TRs) and deiodinase 3, was observed in zebrafish exposed to THs and their analogues.^2,5^ In general, xenogenous compounds can hardly disrupt the thyroid system of living organisms since these have a feedback function in the hypothalamic–pituitary–thyroid axis. However, the abovementioned reports indicate that exogenous THs and their analogues can act as thyroid agonists.

Sewage treatment plants (STPs) are the principal source of environmental release of chemical compounds that can affect both human and wildlife health by acting on the endocrine system, including the TRs.^6,7^ Many studies have reported TR-agonist activity in STP effluents;^8−11^ they suggested that compounds with TR-agonist activity can remain after sewage treatment or be newly originated in that process. Thyroid system disruption has been observed in fish exposed to relatively low concentrations (<3% (v/v)) of STP effluents.^12^ Various studies have indicated that the current influent treatment approaches in STPs cannot effectively eliminate the TR-agonist activity.^10,11,13^ As mentioned above, regular activity of the thyroid system is essential for humans and wildlife; however, environmental water contaminated by STP effluents would affect it because of the incorrect treatment of STP influents in terms of TR-agonist activity. This suggests that the aquatic organisms living near STPs are constantly exposed to the disruption risk of their thyroid system, which would eventually affect human health as well. Therefore, the issue of TR-agonist activity in STP effluents must be immediately addressed to protect both human and wildlife health. It may be resolved by either identifying the compounds having TR-agonist activity or developing a new treatment approach to effectively remove such TR agonists from STP influents. However, the investigation of neither of these solutions has been reported so far probably because most studies focused on the estrogenic activity in STP effluents and influents, while endocrine-disrupting activities in environmental water have been considerably studied.^14−19^

Trace amounts of T4 have been identified in the influents and effluents of an STP.^20^ Nonetheless, the determination of compounds with TR-agonist activity in environmental water was limited to only the report; furthermore, the T4 concentration did not indicate enough contribution to the TR-agonist activity in STP effluents. The present study addressed the issue, the unidentified contribution to TR-agonist activity in STP effluents, by identifying an unknown compound having TR-agonist activity in the STP effluents by using liquid chromatography–tandem mass spectrometry (LC–MS/MS) and liquid chromatography–quadrupole time-of-flight mass spectrometry (LC–QToF–MS). In our previous study,^21^ we evaluated the human TR (hTR) agonist activity of 802 compounds via a yeast two-hybrid assay (TR yeast cell assay), and 17 of them exhibited hTR-agonist activity. In addition, the 802 compounds investigated would cover many suspected endocrine-disrupting chemicals with TR-agonist activity because we included all those having such an activity published by the Endocrine Disruptor Screening Program for the 21st Century (EDSP21) and the Extended Tasks on Endocrine Disruption 2016 project. Therefore, any of the abovementioned 17 compounds may exist as the unknown compound with TR-agonist activity in STP effluents. We attempted to identify 13 of them in STP effluents by chemical analyses. Moreover, we evaluated whether the unknown compound plays an important role in the TR-agonist activity in environmental water by comparing the identification results and hTR-agonist activities in the same effluents through a TR yeast cell assay.

## Materials and Methods

### Reagents

The solvents were of the grade used for testing pesticide residues and polychlorinated biphenyls (Nacalai Tesque, Inc., Japan, and FUJIFILM Wako Pure Chemical Corporation, Japan). The following native standards were obtained: T3 and T4 from Nacalai Tesque, Inc. (Japan); tetrabromobisphenol A (TBBPA), tetrachlorobisphenol A (TCBPA), TRIAC, and 3,3′,5,5′-tetraiodothyroacetic acid (TETRAC) from Tokyo Chemical Industry Co., Ltd. (Japan); 3,3′,5′-triiodo-l-thyronine (rT3), *N*-acetyl l-thyroxine (acetyl-T4), 3,5-diiodothyropropionic acid (DITPA), and 3-chloro-3′,5,5′-triiodo-l-thyronine (3-Cl-T3) from Toronto Research Chemicals, Inc. (Canada); sobetirome (GC-1), 3,3′,5-triiodo-l-thyronine-13C6 (T3-13C6), and l-thyroxine-13C6 (T4-13C6) from Sigma-Aldrich (Missouri, USA); triclabendazole from FUJIFILM Wako Pure Chemical Corporation (Japan); and 3-iodo-l-thyronine (T1) from Santa Cruz Biotechnology, Inc. (Texas, USA). The stock solution of each chemical was prepared with methanol (MeOH) and stored at −30 °C in the dark.

### Sample Collection and Pretreatment

**Figure 1:**
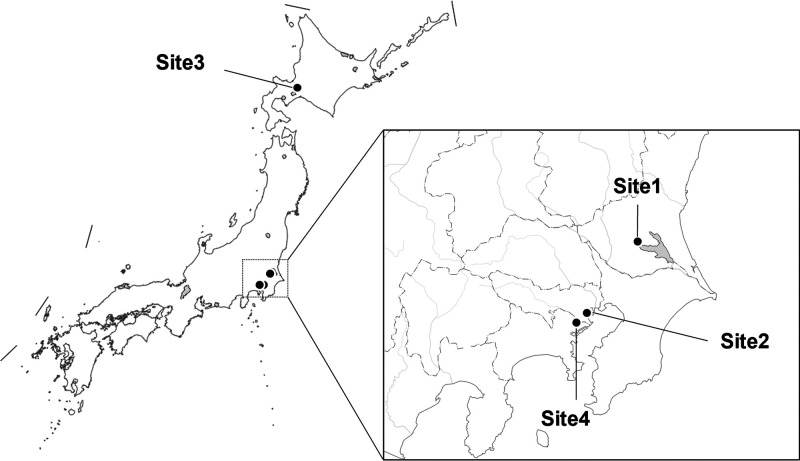
Sampling locations of the environmental waters. Copyright. 2019, Geospatial Information Authority of Japan (GSI). A white map of GSI vector reproduced with permission from GSI was modified in this study.

Environmental samples were collected from downstream of the STPs in the Japanese cities of Tsuchiura (Site1), Minato (Site2), and Sapporo (Site3) because it was determined in our previous studies that these STP effluents in Japan showed relatively strong TR-agonist activity (data not published). Drainage gutter gate water not including sewage was also collected as a reference sample from Kawasaki city in June 2021 (Site4) (Figure 1 and Tables S1 and S2). The Site1 samples were collected from June 2020 to May 2021. The Site2 samples were collected in June (Site2_Jun.) and September (Site2_Sep.) 2021. Three Site3 sample types were collected in June 2021 and May 2022: in the STP effluent (Site3_EF), in the water before chlorination but after activated sludge treatment (Site3_AS), and in the STP influent (Site3_IF). From each final STP effluent sample (3.0 L), a sample solution (1.0 L) was glass-filtered to remove insoluble particulate matter; then, the solution was concentrated using a solid-phase extraction disk (3M Empore SDB-XD, 2242), and the concentrated compounds were eluted with MeOH (5.0 mL) and dried under N_2_ purge. Three or more dried samples were prepared from each sewage sample. Next, the sample was dissolved in 100 or 500 μL of MeOH for, respectively, the TR yeast cell assay, to estimate the hTR-agonist activity, or the chemical analysis via LC–MS/MS, to identify the 13 compounds having hTR-agonist activity. Table S3 lists the recovery rates of the 13 compounds by this pretreatment.

### Yeast Two-Hybrid Assay

We used a yeast two-hybrid TH assay system utilizing yeast cells (*Saccharomyces cerevisiae* Y190) where the hTR alpha and its coactivator, that is, the transcriptional intermediary factor 2, were introduced. The conjugation was adapted to a chemiluminescent reporter gene assay (for β-galactosidase) with a 96-well culture plate.^22−24^ The yeast cells were preincubated for 24 h at 30 °C under shaking in a modified SD medium (lacking tryptophan and leucine, 0.88% dextrose), and the cell density was adjusted to an absorbance of 1.75–1.84 at 595 nm. The medium (60 μL, containing 0.2% dimethyl sulfoxide (DMSO)) was placed in the all wells of a black 96-well culture plate for chemiluminescence measurements. A test solution (20 μL) (e.g., environmental samples and test chemicals) was added to 480 μL of the medium, and the aliquots of this mixture (60 μL) were also added to the wells of the first row. This test solution was serially diluted from rows 1 to 7 (each in duplicate); then, a yeast cell suspension (60 μL) was also added to all wells (including those in row 8, which served as the solvent control). Thus, the first row contained a 10,000 nΜ solution of the target chemical, the second row a 2500 nM solution of it, and so on. After the addition of the yeast suspension and vortex mixing, the plates were incubated at 30 °C under high humidity for 4 h. A lysis solution (50 μL), prepared using 2.0 mg of Zymolyase 100T (from *Arthrobacter luteus*; Nacalai Tesque, Inc., Japan) in 7 mL of a buffer solution, was added to each well of the plate for enzymatic digestion. The plate was then incubated at 37 °C for 1 h after agitation. A total of 80 μL of a Tropix Gal-Screen substrate and a Sapphire-II enhancer (Applied Biosystems, Massachusetts, USA) solution in phosphate buffer, for inducing chemiluminescence from the released β-galactosidase, was added to each well, followed by incubation at 30 °C for 10 min after agitation. Afterward, the plate was placed in a 96-well plate luminometer (Luminescencer JNR AB2100, Atto Corp., Japan), and the chemiluminescence produced by β-galactosidase in each well was measured. The agonist activity was evaluated as the 10-fold effective concentration (EC_×10_), which is the test solution concentration that produces a chemiluminescent signal intensity 10 times that of the blank control, that is, the pure DMSO aqueous solution. The agonist activity was derived from the linear regression of the dose–response curve, which was obtained by plotting the luminescence intensity against the test solution concentration. The hTR-agonist activity of the test solution exhibiting it in the first assay was evaluated with three replicates on different days to ensure precision.

### Fractionation Experiment

An Oasis HLB Plus LP extraction cartridge (Waters Corporation, Massachusetts, USA) was used to fractionate STP samples. A N_2_-purged STP sample dissolved in 500 μL of purified water was loaded into the HLB column and eluted stepwise by 10–100% MeOH. The hTR-agonist activity of the sample was evaluated by the TR yeast cell assay and LC–MS/MS.

### Deconjugation Treatment

An STP sample (100 mL) was filtered using a Ø 47 mm glass filter (Whatman GF/C, 47 mm). A 10 mL aliquot of the filtered sample was pretreated prior to chemical analysis by LC–MS/MS (raw sample). The remaining filtered sample (90 mL) was subjected to deconjugation treatment. Briefly, sodium acetate (100 mg) was added to the sample, and acetic acid was added to adjust the pH to 4.8–5.0. To a 10 mL aliquot of the pH-adjusted sample, β-glucuronidase/arylsulfatase liquid enzyme (100 μL) from *Helix pomatia* (β-glucuronidase and sulfatase activities of ∼100,000 and ∼47,500 units/mL, respectively, from Roche Diagnostics GmbH, Mannheim, Germany) was added. The resulting sample was incubated at 37 °C for 18 h. After incubation, the sample was pretreated prior to chemical analysis via LC–MS/MS (enzyme sample).

### Instruments

An LC–MS/MS system (Triple Quad 5500+, QTRAP Ready, AB Sciex, Japan) was utilized for identifying and quantifying the 13 compounds with hTR-agonist activity. The general conditions were as follows: mobile phase (MeOH/0.5 mM ammonium fluoride in water); 20% MeOH (0–1 min), 20–50% MeOH linear gradient (1–10 min), 50–90% linear gradient (10–25 min), and 90% MeOH (25–35 min); a flow rate of 0.2 mL/min; an InertSustain C18 column with an inner diameter of 2.1 mm and a length of 150 mm (GL Sciences, Japan); a temperature of 40 °C; detection via electrospray ionization (ESI) in the negative ion mode with multiple reaction monitoring (MRM, Table S4); and an injection volume of 6.0 μL. The following MS settings were adopted: curtain and collision gas pressures of 40 and 8 psi, respectively; ion spray voltage of −4500 V; temperature of 500 °C; and ion source gas 1 and 2 pressures of 80 and 70 psi, correspondingly. The instrumental detection limits of this system for the 13 target compounds were evaluated (Table S5); the limits of quantification (LOQ) were also calculated, and their values were specified in the captions of Tables S6 and S7.

The LC–QToF–MS apparatus (Agilent) consisted of a 1260 Infinity liquid chromatographer and a 6546 QToF–MS system. The operating conditions were as follows: mobile phase (MeOH/0.5 mM ammonium fluoride in water); 20% MeOH (0–1 min), 20–50% MeOH linear gradient (1–10 min), 50–90% linear gradient (10–25 min), and 90% MeOH (25–35 min); a flow rate of 0.2 mL/min; an InertSustain C18 column with an inner diameter of 2.1 mm and a length of 150 mm (GL Sciences, Japan); a temperature of 40 °C; detection via negative ESI; and an injection volume of 1.0 mL. The following MS settings were used: a gas temperature of 320 °C, a drying gas flow of 8 L/min, a nebulizer pressure of 35 psi, a sheath gas temperature and flow of 350 °C and 11 L/min, respectively, a nozzle voltage of 1.0 kV, a vcap voltage of 3500 V, and a mass range selection of 100–1100 (*m*/*z*).

### Calculation of Rate of Contribution to the hTR-Agonist Activity

The contribution rate of each investigated compound to hTR-agonist activity in the STP and reference effluents was calculated as follows:
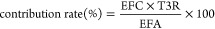
1where EFC is the compound concentration (in ng/L) in the effluents quantified by LC–MS/MS, T3R is the ratio between the hTR-agonist activities of the target compound and T3, the values of which will be published in another study of ours,^21^ and EFA is the overall hTR-agonist activity (in ng-T3 eq/L) in the STP effluents and the reference water.

## Results and Discussion

### Determination via LC–MS/MS of the 13 Compounds in the STP Effluent Samples

The 13 compounds with hTR-agonist activity were analyzed using LC–MS/MS (Tables S6 and S7), and 11 of them were identified at least once in the STP effluent samples, while acetyl-T4 and DITPA were never detected in this study. TBBPA and TRIAC were found in all the samples with concentration ranges of 0.032–0.15 and 0.30–4.2 ng/L, respectively; TCBPA and T1 were also detected in most samples with concentration ranges of 0.022–0.19 and 0.00024–0.50 ng/L, correspondingly. Moreover, 3-Cl-T3, a chlorinated derivative of rT3, was identified in the Site4, Site2_Sep., and a few Site1 samples; especially, it showed the highest concentration in all the Site4 samples (24 ng/L). T3, rT3, and T4 were also detected in several samples, but their concentrations were extremely low (<0.5 ng/L). The two bisphenol derivatives, TBBPA and TCBPA, were often identified in the environmental water samples;^25,26^ however, research on THs and their analogues in the environment was limited to the report below. A previous study has detected T4 in STP influents and effluents.^20^ Therefore, the detection of THs and their analogues in the environment was demonstrated for the first time in this study.

In our previous study, we suggested that TRIAC, TETRAC, and GC-1 could strongly cause hTR-agonist activity.^21^ This study identified TRIAC in all the STP effluent samples, suggesting that it might closely relate to the hTR-agonist activity in the environment. Moreover, it was reported that an EC_80_ of TRIAC is 6 nM (3726 ng/L) to zebrafish,^2^ but EC_×10_ was 0.008 nM to hTR (4.9 ng/L).^21^ The EC_×10_ value is close to the TRIAC concentrations detected in this study, and it was reported that the zebrafish genome shares 71% homology with humans.^27^ In addition, 10 nM T3 affected the ontogenetic expression of TH signaling genes in developing zebrafish,^5^ and the T3 concentration may be equivalent to 32 ng/L TRIAC because TRIAC has a 200 times higher hTR-agonist activity compared to T3.^21^ These studies suggest that the environmental concentrations of TRIAC may affect the thyroid system of aquatic organisms. Therefore, we tried to define the hTR-agonist activity in the environment based on the relation between the LC–MS/MS identification results for the 13 compounds and the hTR-agonist activity of the STP effluents assessed via the TR yeast cell assay.

### Determination of the Contributions of the 13 Compounds to the hTR-Agonist Activity in the STP Effluents

The hTR-agonist activity of the STP effluents was evaluated by the TR yeast cell assay (Table S8). Both the Site2 samples exhibited relatively high hTR-agonist activities (721 and 533 ng-T3 eq/L, respectively), while the Site4 sample did not show the hTR-agonist activity. Furthermore, the annual behavior of the hTR-agonist activity in the Site1 samples fluctuated between 108 and 308 ng-T3 eq/L. The TR-agonist activity range in STP effluents worldwide is 1–204 ng-T3 eq/L^9−11^ therefore, the values measured in the present study are about 3–700 times higher than those reported in previous studies.

The total contribution rate (TCR) to the hTR-agonist activity by the 13 compounds identified in the STP effluents was calculated (Tables 1 and 2). For the Site1 samples, it was 60–148%, and TRIAC contributed to more than 99% of the TCR among all of them. In the Site2_Jun., Site2_Sep., and Site3_EF samples, the TCR was 124, 110, and 111%, respectively, and TRIAC correspondingly contributed to 119, 100, and 94% of it. The hTR-agonist activity in those STP effluents, evaluated by the TR yeast cell assay, was almost consistent with the LC–MS/MS identification results; TRIAC averagely contributed to ∼100% of their activity. TRIAC is as a metabolite of T3 and T4; moreover, its half-life is short.^28,29^ Based on previous findings, the TRIAC detection in this study was unexpected; hence, further confirmation of its presence in STP effluents is required. On the other hand, except TRIAC, all the TCRs in the Site1 samples were below 1%, and those in the Site2 ones ranged from 4 to 10%, of which TETRAC was the main contributor. The Site3_EF sample, conversely, showed a relatively high contribution (17%) without TRIAC; the main contributor was GC-1, which exhibited a high hTR-agonist activity following TRIAC and TETRAC in our previous study.^21^ Furthermore, the TCRs for the Site1 samples collected in June 2020 and September 2021 were relatively low that TRIAC in both of them contributed to 60% of their hTR-agonist activities. These results suggest that there are contributors other than TRIAC to the hTR-agonist activity in the environment. However, these contributors were unknown compounds in the case of the two Site1 samples mentioned above. They were probably excluded from not only the 13 compounds investigated here but also the list of compounds with rat TR-agonist activity published by the EDSP21^30^ since all the latter were evaluated in our previous study.^21^

**Table 1:** Contribution Rates of the 13 Compounds to the hTR-Agonist Activities in Site1 (%)^a^

(%)	Jun.	Jul.	Aug.	Sep.	Oct.	Nov.
TRIAC	61	87	102	60	123	96
TETRAC	-	-	-	-	-	-
GC-1	-	-	3.5 × 10^–2^	-	-	-
T3	3.3 × 10^–3^	1.5 × 10^–2^	1.5 × 10^–2^	4.2 × 10^–3^	2.3 × 10^–2^	-
T4	-	-	-	-	-	-
DITPA	-	-	-	-	-	-
3-Cl-T3	-	8.8 × 10^–3^	-	-	-	-
rT3	-	3.7 × 10^–3^	1.8 × 10^–3^	2.4 × 10^–3^	2.6 × 10^–3^	-
acetyl T4	-	-	-	-	-	-
T1	3.2 × 10^–3^	3.1 × 10^–3^	4.7 × 10^–4^	1.0 × 10^–6^	4.7 × 10^–4^	1.6 × 10^–4^
TCBPA	4.9 × 10^–4^	5.1 × 10^–4^	7.4 × 10^–4^	4.3 × 10^–4^	5.3 × 10^–4^	4.0 × 10^–4^
TBBPA	1.1 × 10^–4^	1.2 × 10^–4^	1.9 × 10^–4^	1.2 × 10^–4^	1.4 × 10^–4^	2.1 × 10^–4^
triclabendazole	7.5 × 10^–7^	3.5 × 10^–5^	2.2 × 10^–5^	7.5 × 10^–7^	3.2 × 10^–5^	-
TCRs	61	87	102	60	123	96

(%)	Dec.	Jan.	Feb.	Mar.	Apr.	May
TRIAC	148	92	116	136	106	79
TETRAC	-	-	-	-	-	-
GC-1	-	-	-	-	-	-
T3	-	1.2 × 10^–2^	1.6 × 10^–2^	-	-	0.18
T4	-	1.2 × 10^–2^	-	-	-	3.8 × 10^–2^
DITPA	-	-	-	-	-	-
3-Cl-T3	6.1 × 10^–2^	1.5 × 10^–2^	-	-	6.4 × 10^–2^	-
rT3	3.9 × 10^–3^	5.8 × 10^–3^	8.4 × 10^–3^	-	6.9 × 10^–3^	-
acetyl T4	-	-	-	-	-	-
T1	3.5 × 10^–3^	-	2.6 × 10^–3^	1.9 × 10^–3^	2.8 × 10^–3^	-
TCBPA	5.5 × 10^–4^	4.5 × 10^–4^	8.6 × 10^–4^	-	8.6 × 10^–4^	2.2 × 10^–4^
TBBPA	3.0 × 10^–4^	1.8 × 10^–4^	1.5 × 10^–4^	9.7 × 10^–5^	9.1 × 10^–5^	7.7 × 10^–5^
triclabendazole	-	-	-	5.4 × 10^–6^	3.8 × 10^–6^	-
TCRs	148	92	116	136	106	79

a All values indicate the contribution rate of each compound to the hTR-agonist activity in the STP effluents by the TR yeast cell assay. Hyphen means not detected by LC–MS/MS, and the maximum contribution rates calculated from LOQ (%) are given below: TRIAC = 0.43, TETRAC = 0.25, GC-1 = 6.9 × 10^–2^, T3 = 2.1 × 10^–3^, T4 = 2.1 × 10^–3^, DITPA = 7.9 × 10^–4^, 3-Cl-T3 = 1.2 × 10^–3^, rT3 = 6.7 × 10^–4^, acetyl T4 = 2.0 × 10^–4^, T1 = 2.6 × 10^–5^, TCBPA = 1.3 × 10^–5^, TBBPA = 8.9 × 10^–6^, and triclabendazole = 5.2 × 10^–7^. All values are represented as the mean of three replicates (*n* = 3). The concentrations in the STP effluents quantified by LC–MS/MS are shown in Table S6 (ng/L). The hTR-agonist activities in Site1 are shown in Table S8. A comparison with the 13 compound concentrations as T3 equivalent and the hTR-agonist activities is given in Table S9 (ng-T3 eq/L). TCR is the total contribution rate (%). The dose–response and standard curves by the yeast assay and LC–MS/MS, respectively, are shown in Figure S1.

**Table 2:** Contribution Rates of the 13 Compounds to the hTR-Agonist Activities in Site2 and Site3 (%)^a^

(%)	site2_Jun.	site2_Sep.	site3_EF
TRIAC	119	100	95
TETRAC	4.4	10	
GC-1			17
T3		7.1 × 10^–2^	
T4	1.5 × 10^–2^	7.7 × 10^–2^	8.3 × 10^–2^
DITPA			
3-Cl-T3		3.3 × 10^–2^	
rT3		2.1 × 10^–2^	
acetyl T4			
T1		4.0 × 10^–4^	
TCBPA	1.5 × 10^–4^	1.4 × 10^–4^	3.3 × 10^–4^
TBBPA	6.2 × 10^–5^	5.5 × 10^–5^	5.9 × 10^–4^
triclabendazole			
TCRs	124	110	111

a Hyphen refers to not detected by LC–MS/MS, and the maximum contribution rates calculated from LOQ (%) are given below: TRIAC = 0.43, TETRAC = 0.25, GC-1 = 6.9 × 10^–2^, T3 = 2.1 × 10^–3^, T4 = 2.1 × 10^–3^, DITPA = 7.9 × 10^–4^, 3-Cl-T3 = 1.2 × 10^–3^, rT3 = 6.7 × 10^–4^, acetyl T4 = 2.0 × 10^–4^, T1 = 2.6 × 10^–5^, TCBPA = 1.3 × 10^–5^, TBBPA = 8.9 × 10^–6^, and triclabendazole = 5.2 × 10^–7^. All values are represented as the mean of three replicates (*n* = 3). These concentrations in the STP effluents quantified by LC–MS/MS have been shown in Table S7 (ng/L), and the hTR-agonist activities in the sites have been shown in Table S8. A comparison with the 13 compound concentrations as T3 equivalent and the hTR-agonist activities is shown in Table S10 (ng-T3 eq/L). TCR is the total contribution rate (%). The dose–response and standard curves by the yeast assay and LC–MS/MS, respectively, are shown in Figure S1.

The Site4 sample showed a different trend. It did not exhibit hTR-agonist activity in the TR yeast cell assay, and TRIAC was not identified. In contrast, 3-Cl-T3 with a relatively high hTR-agonist activity was detected; therefore, this result is inconsistent with the TR yeast cell assay, which showed the no hTR-agonist activity. However, the 3-Cl-T3 concentration in the Site4 sample was only 24 ng/L, and the hTR-agonist activity as predicted from the LC–MS/MS results was ∼8.5 ng-T3 eq/L. Therefore, this undetectable activity may be due to the performance of the TR yeast cell assay, although it was not a surprise since the Site4 sample is not an STP effluent. The identification of 3-Cl-T3, instead, was unexpected. In any case, the hTR-agonist activity of the Site4 sample was lower than that of the STP effluent samples, and it would relate to the existence of TRIAC.

### Confirmation of TRIAC Presence by LC–QToF–MS

QToF–MS is a useful tool for the precise identification of compounds in environmental samples.^31^ Therefore, the TRIAC identified in the STP effluent samples was confirmed by a difference (mass error) in its exact mass, a theoretical value derived from a molecular formula, and the accurate mass of compounds like TRIAC (ClT) in the STP effluents, estimated by LC–QToF–MS. Its precise identification was conducted as follows. First, the ClT in the STP effluents was measured for comparison between accurate and exact mass. Next, the retention time (RT) in the TRIAC standard solution (TSS) was compared with that of the ClT in the Site2_Sep. sample. Finally, the Site2_Sep. sample spiked TSS was analyzed to confirm an overlap between the ClT and TRIAC standard peaks.

**Figure 2:**
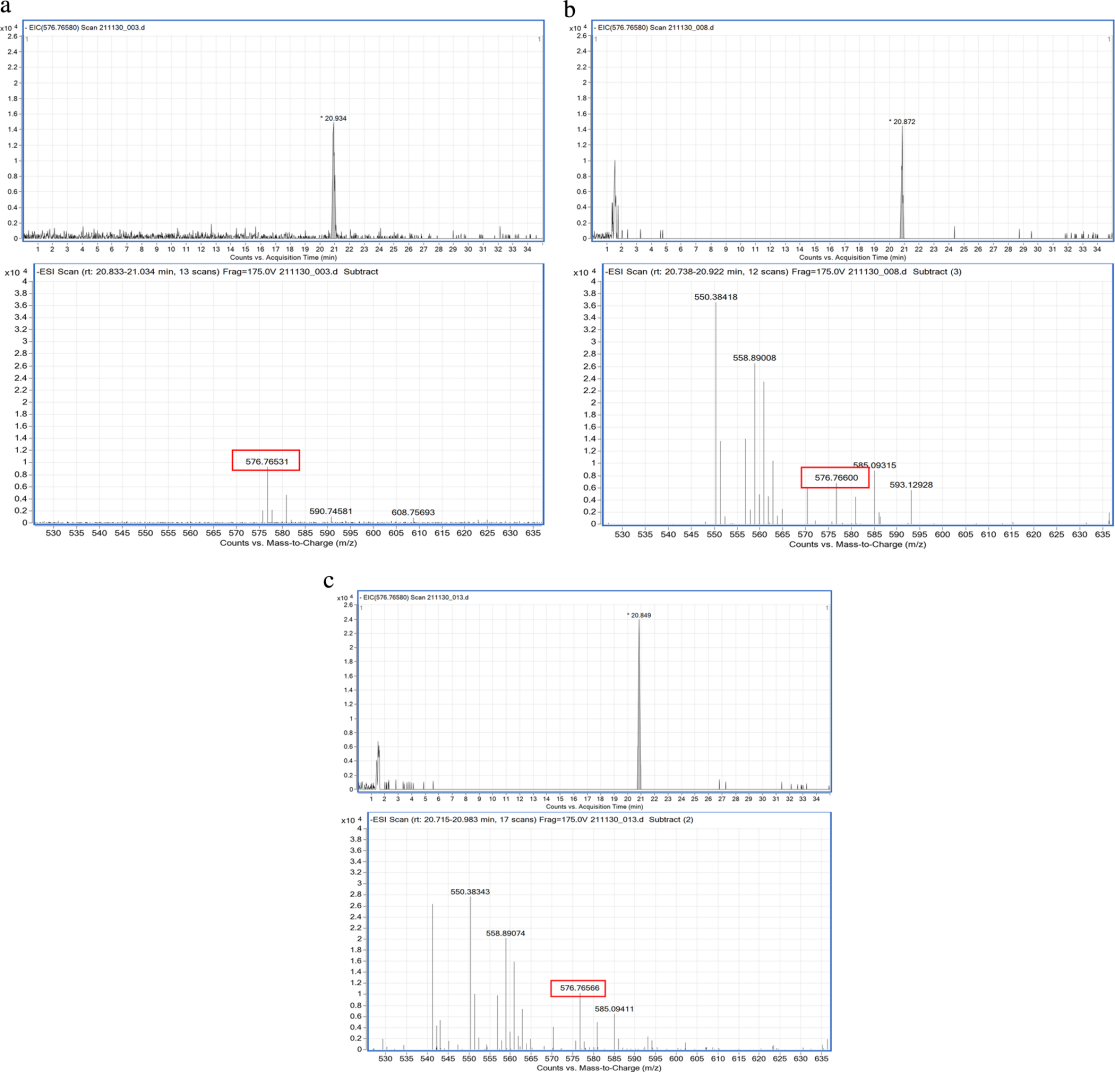
Results of LC–QToF–MS analysis: (a) TRIAC standard in methanol, (b) compound like TRIAC (ClT) in the sample collected in Minato city (Site2_Sep.), and (c) ClT-spiked TSS in the Site2_Sep. sample.

TRIAC was identified as a negative ion of M-COOH in this study because its peak intensity was extremely stronger than the M-H one. Therefore, the mass errors were calculated using the exact mass of M-COOH and the accurate masses of the ClT. The mass errors in the Site2_Sep. sample were within ±1 ppm (Table 3; those mass spectra are shown in Figure S2). Compounds with a mass error within ±10 ppm are probably positive compounds.^31−34^ The peaks in the TSS and Site2_Sep. samples were measured at the same RT (Figure 2a,b); in addition, the peaks of the ClT and TRIAC standard spiked in the Site2_Sep. sample were overlapped (Figure 2c). The overlapped peak height obviously increased compared with the single peaks in the TSS and Site2_Sep. sample. If the Site2_Sep. sample did not include TRIAC, the two peak tops of the ClT and TRIAC standard spiked would have appeared in the overlapped peak, and its height would not change compared with the single peaks in the TSS and Site2_Sep. sample. Therefore, these results demonstrate that the ClT in the Site2_Sep. sample was TRIAC. Moreover, each ClT in the Site1 and Site2_Jun. samples also had mass errors between −0.87 and 20 ppm. All the Site1 samples, except that collected in April, showed mass errors within ±10 ppm. However, the mass error in agricultural chemicals by LC–ToF–MS is usually within 38 ppm.^35^ Furthermore, the accurate masses obtained via TOF mass spectrometry might depend on machine conditions such as the instrument temperature.^36^ Therefore, all the ClTs in the Site1 and Site2_Jun. samples could also be TRIAC.

**Table 3:** Exact Mass of TRIAC vs the Accurate Masses by LC–QToF–MS in the Site2_Sep Sample^a^

exact mass (M-COOH)	accurate mass (M-COOH)	Δ value (exact – accurate)	mass error (ppm)
576.7658	576.7658 (±0.00017)	0.0002 (±0.000047)	0.3 (±0.082)

a Exact mass means a theoretical value calculated from a molecular formula. Accurate mass is acquired by measurement using QToF–MS. Δ value is the difference between the exact mass and the accurate mass. Mass error is parts per million (ppm) of the Δ value. All values except for the exact mass are represented as the mean of three replicates (*n* = 3). M is a molecule; it is TRIAC (C_14_H_9_I_3_O_4_ = 621.7635). “M-COOH” means desorption of “COOH” from a molecule. Each mass spectrum including the accurate mass has been shown in Figure S2.

In addition, the validity of TRIAC was confirmed by fractionation experiments on the Site1_Sep. sample using a HLB column (Table S11). The TRIAC and the hTR-agonist activity were detected only in the 90 and 100% MeOH fraction samples. Their behavior corresponded with the MeOH ratio at the RT of TRIAC by the LC–MS/MS method used in this study. On the other hand, the moderate TRIAC contribution to the hTR-agonist activities found by the assay also correspond with the result in Table 1. This was because the Site1 sample, which was collected during September 2020, was used in this fractionation experiment. The Sep. sample showed the strongest TR-agonist activity among the Site1 samples; however, the contribution of TRIAC was only ∼60% of the TR-agonist activity. Therefore, there are unknown contributors other than TRIAC. The fractionation results also supported the presence of the unknown contributors.

### Origin of TRIAC in the STP Effluents

**Figure 3:**
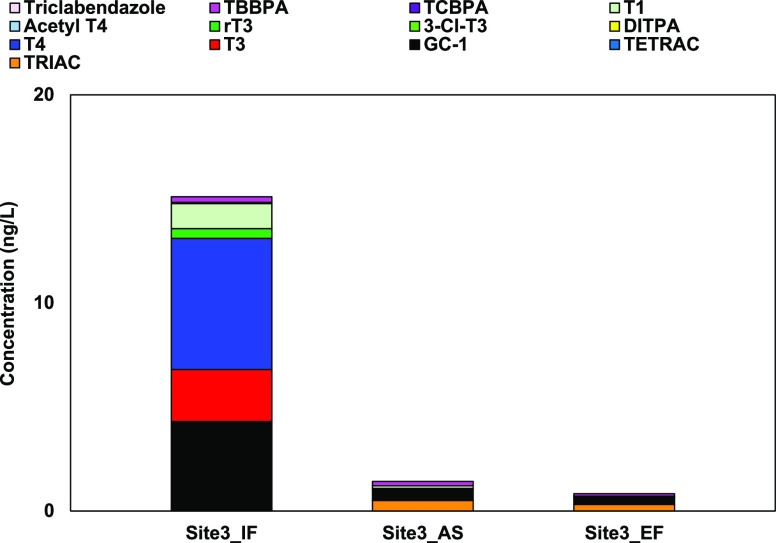
Difference between the influent (Site3_June_IF), after active sludge treatment but before chlorination (Site3_June_AS), and effluent (Site3_June_EF) samples collected from the STP in Sapporo city in terms of the concentration of the 13 target compounds by LC–MS/MS. All values were calculated as the mean of three replicates.

The 13 compounds in the Site3 samples were analyzed by LC–MS/MS to determine the relation between TRIAC production and sewage treatment in the plants (Figure 3). TRIAC, interestingly, was not detected in the influent (Site3_June_IF), while it was found in the effluent (Site3_June_EF) and the Site3_June_AS samples. On the other hand, relatively high T3 and T4 concentrations (2.5 and 6.3 ng/L, respectively) were identified in the June_IF sample, but their values dramatically decreased in the June_AS and June_EF ones, where T3 was not found at all and the T4 concentrations were approximately one-hundredth of that in the June_IF sample. These results suggested that TRIAC in the June_EF and June_AS samples may be a degradation product of T3 and T4 in the June_IF one since TRIAC is usually generated from them.^28,29^ The TRIAC concentration in the June_EF sample is similar to that in the June_AS samples. Therefore, the activated sludge treatment may be closely related to the TRIAC production in STPs. Nonetheless, further study on TRIAC in STP influents and effluents is required because in this study, the samples from STP influents and after activated sludge treatment were collected only from the STP in Sapporo city. However, if our hypothesis about TRIAC is correct, some behavior of TR activity in the environment can be explained well.

The efficiency of TR activity removal in STPs might be inadequate because the TR-agonist activity has been reported for many STP effluents,^10,11,13,37^ whereas the estrogen activity in STP influents is efficiently removed by the sewage treatment.^9,19^ Moreover, in the present study, the hTR-agonist activity during summer (June–September) exhibited an increasing trend (Table S8). It was reported that the sewage treatment in summer achieved sufficient removal of all the compounds having estrogen activity, while it was insufficient in winter.^38^ These behaviors might also be associated with biodegradation performance due to temperature changes of the sewage during the activated sludge treatment;^38,39^ in other words, the activated biodegradation would generate TR-agonist activity in the STP effluents because TRIAC is a metabolite of T3 and T4. However, it can remove estrogen activity in STP influents since its main source is 17β-estradiol rather than the metabolites.^18^ On the other hand, biodegradation in winter would be relatively weak compared with that in summer,^38,39^ and this difference would lead to the relatively low TR-agonist activity in winter because the temperature change would weaken the T3 and T4 degradation in the STP influents. These phenomena might indicate insufficient removal efficiency of the sewage treatment in STPs. Furthermore, it was suggested that the TR activity in the environment could be related to some metabolites.^40^

**Figure 4:**
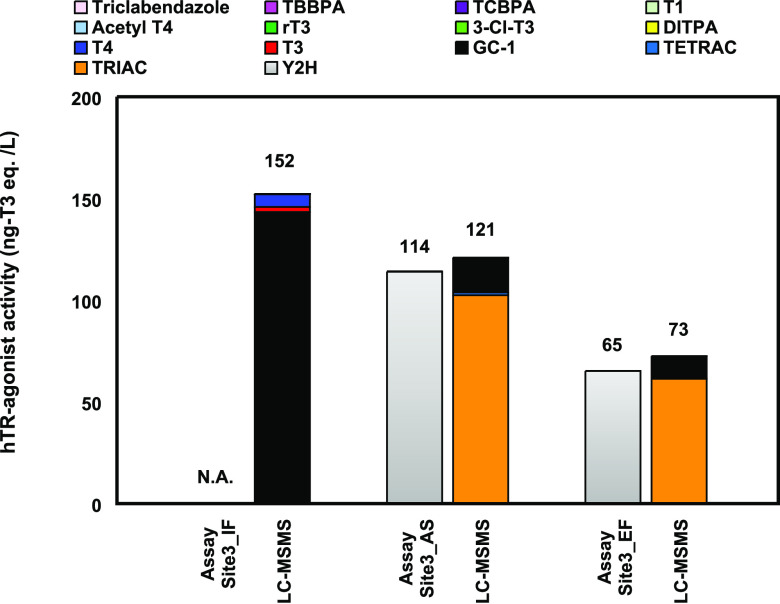
hTR-agonist activity determined by the TR yeast cell assay and compound identification by LC–MS/MS of the STP samples collected in the Sapporo city during June 2021 (Site3_June). N.D. means not detected. All the values were expressed as the T3 equivalent concentration (ng-T3 eq/L) and calculated as the mean of three replicates. N.A. means no activity.

The hTR-agonist activities of the June_EF, June_AS, and June_IF samples were evaluated by the TR yeast cell assay (Figure 4), but the June_IF sample showed no activity. This may be due to another activity in the STP influents, such as the TR antagonist activity.^11,13,37,40−42^ Therefore, the hTR-agonist activity of the June_IF sample was defined as 152 ng-T3 eq/L, as predicted from the result by LC–MS/MS in Figure 4. The hTR-agonist activity of the June_IF sample was higher than those of the June_EF and June_AS ones, which could be attributed to the presence of GC-1. The GC-1 activity was 143 ng-T3 eq/L in the June_IF sample and about one-tenth of it in the June_EF and June_AS ones (11 and 17 ng-T3 eq/L, respectively). However, the hTR-agonist activities of the June_EF and June_AS samples retained about 50–80% of that in the June_IF one despite the decrease in the GC-1 concentration. This is probably due to the TRIAC production because TRIAC has a six times higher hTR-agonist activity than GC-1.^21^ If TRIAC in STP effluents is produced from T3 and T4 in the STP influents, its concentration would depend on those of T3 and T4. Therefore, the hTR-agonist activity in STP effluents may exceed that in STP influents depending on the T3 and T4 concentrations.

**Figure 5:**
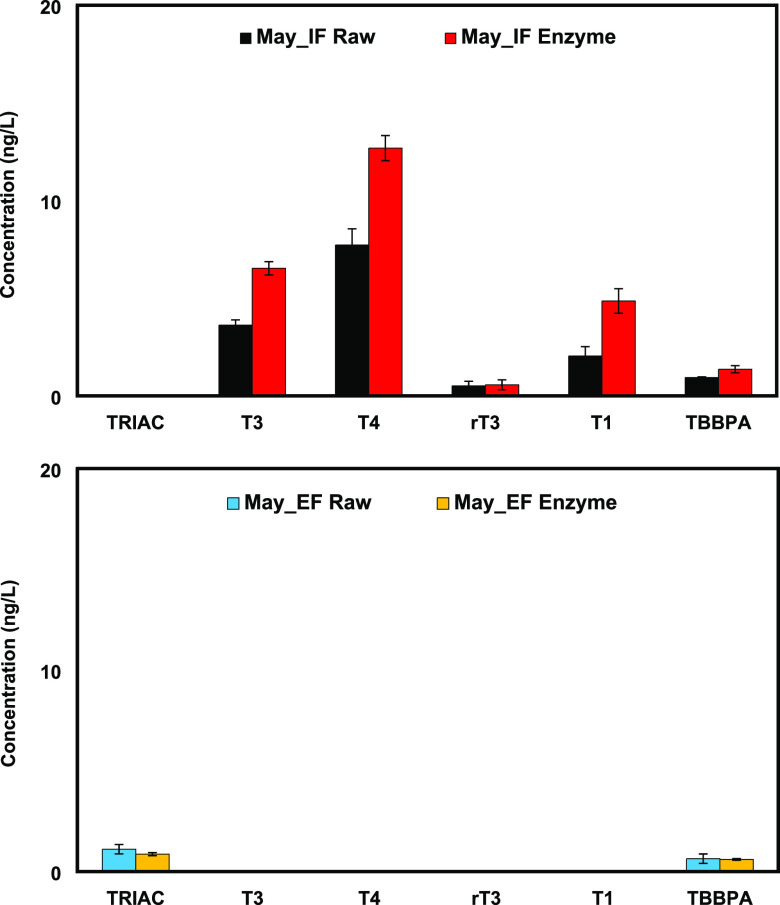
Evaluation of the TH and the analogue conjugations by LC–MS/MS in STP samples collected in the Sapporo city during May 2022 (Site3_May). Raw sample was not deconjugated. The enzyme-treated sample was deconjugated using β-glucuronidase/arylsulfatase liquid enzyme. The error bar represents standard errors (*n* = 3).

It has been suggested that TRIAC is efficiently glucuronidated in human and rat.^43,44^ Therefore, the conjugate in the STP samples (Site3_May_IF and EF) was evaluated. The STP samples were deconjugated according to previous studies^45−47^ and evaluated by comparing the TRIAC concentration in raw (no enzyme treatment) and enzyme-treated samples. TRIAC was not detected in both raw and enzyme-treated samples of May_IF. It was identified in both raw and treated samples of May_EF; however, the concentrations found in the samples were the same. These results supported the hypothesis that the origin of TRIAC is from the degradation of T3 and T4. On the other hand, the concentrations of T1, T3, and T4 in the samples of May_IF increased after the enzyme treatment. This indicated that THs and some of their analogues existed potentially as both free ion and conjugated forms in an STP influent. Nonetheless, the conjugates were not an issue since the three relevant compounds were removed completely in the effluent. Moreover, an interesting behavior was observed in Figure 5. In the May_EF sample, ∼1.0 ng/L TRIAC was detected, which was approximately equal to the predicted hTR-agonist activity of 200 ng-T3 eq/L. The activity was ∼20 times stronger than the predicted hTR-agonist activity in the May_IF sample of about 10 ng-T3 eq/L. The reverse phenomenon observed in the samples of May_IF and May_EF was probably due to the high concentrations of T3 and T4 in the influent. An evaluation of TRIAC at relevant environmental concentrations by *in vivo* assays and an appropriate treatment to reduce the activity in sewage are required.

## Supporting Information Available

Geographic coordinates of the sampling sites (Table S1); characteristics of the water samples (Table S2); recovery rates of the 13 compounds by pretreatment (Table S3); MRM method of the 13 compounds using LC–MS/MS (Triple Quad 5500+) (Table S4); instrumental detection limits of the LC–MS/MS for the 13 compounds investigated (Table S5); concentration, measured via LC–MS/MS, of the 13 target compounds in the Site1 samples (Table S6); concentration, measured via LC–MS/MS, of the 13 target compounds in the Site2, 3, and 4 samples (Table S7); hTR-agonist activity in the samples measured by the TR yeast cell assay (Table S8); comparison with concentration expressed as equivalent to T3 of the 13 target compounds and the overall hTR-agonist activity in the Site1 samples (Table S9); comparison with concentration expressed as equivalent to T3 of the 13 target compounds and the overall hTR-agonist activity in the Site2, 3, and 4 samples (Table S10); behavior evaluation of TRIAC and hTR-agonist activity by fractionation using a HLB column (Table S11); dose–response and standard curves by yeast assay and LC–MS/MS, respectively (Figure S1); and mass spectra measured via LC–QToF–MS, including the accurate masses of compounds like TRIAC of the STP effluent collected in Minato (Site2_Sep.) (Figure S2) (PDF)

## Abbreviations

EPA: Environmental Protection Agency
NIES: National Institute for Environmental Studies
RT: retention time
STP: sewage treatment plants
TCR: total contribution rate
TETRAC: tetraiodothyroacetic acid
TH: thyroid hormones
TR: thyroid hormone receptor
TRIAC: triiodothyroacetic acid
TSS: TRIAC standard solution
