# Supplementary material for: Identification by Liquid Chromatography–Tandem Mass Spectrometry and Liquid Chromatography–Quadrupole Time-of-Flight Mass Spectrometry of the Contributor to the Thyroid Hormone Receptor Agonist Activity in Effluents from Sewage Treatment Plants
Source: Environ Sci Technol. 2022 Sep 13;56(19):13709–18. doi: 10.1021/acs.est.2c02648 (PMC9536310; doi:10.1021/acs.est.2c02648)
Supplement: Supplementary file 1 — es2c02648_si_001.pdf [file es2c02648_si_001.pdf]

Supporting Information

Identification by Liquid Chromatography–Tandem Mass Spectrometry and Liquid Chromatography–Quadrupole Time-of-Flight Mass Spectrometry of the Contributor to the Thyroid Hormone Receptor Agonist Activity in Effluents from Sewage Treatment Plants

Ryo Omagari [a*], Mayuko Yagishita [b], Fujio Shiraishi [a], Shoji Nakayama [a], Masanori Terasaki [c], Tetsuya Tanigawa [d], Ichiro Yamauchi [e], Takuya Kubo [f], Daisuke Nakajima [a, g]

[a] Health and Environmental Risk Division, National Institute for Environmental Studies (NIES), Tsukuba-city, Ibaraki, 305-8506, Japan

[b] Department of Life and Environmental Science, Prefectural University of Hiroshima, Shobara-city, Hiroshima, 727-0023, Japan

[c] Graduate School of Arts and Sciences, Iwate University, Morioka-city, Iwate, 020-8550, Japan

[d] Graduate School of Engineering, Kyoto University, Katsura, Nishikyo-ku, Kyoto 615-8510, Japan

[e] Department of Diabetes, Endocrinology and Nutrition, Kyoto University Graduate School of Medicine, 606-8507

[f] Department of Material Chemistry, Graduate School of Engineering, Kyoto University, Katsura, Nishikyo-ku, Kyoto 615-8510, Japan.

[g] Graduate School of Pharmaceutical Sciences, Chiba University, Chiba-city, Chiba, 260-8675, Japan

Corresponding author: Ryo Omagari Ph.D.

*E-mail: omagari.ryo@nies.go.jp

Postal address: Health and Environmental Risk Division, National Institute for Environmental Studies, 16-2 Onogawa, Tsukuba-shi, Ibaraki-ken 305-8506 Japan

Content overview: 13 Pages, 1 Figure, and 10 Tables

Summary

**Table S1.** Geographic coordinates of the sampling sites.	**Page S3**
**Table S2**. Characteristics of the water samples.	**Page S4**
**Table S3**. Recovery rates of the 13 compounds by the pretreatment.	**Page S5**
**Table S4**. MRM of the 13 compounds.	**Page S6**
**Table S5.** Instrumental detection limits of the 13 compounds.	**Page S7**
**Table S6**. Concentration (ng/L) of the 13 compounds in site 1.	**Page S8**
**Table S7**. Concentration (ng/L) of the 13 compounds in site2–site4.	**Page S9**
**Table S8**. hTR-agonist activity observed in the sites.	**Page S10**
**Table S9**. Concentration (ng-T3 eq/L) of the 13 compounds in site1.	**Pages S11–12**
**Table S10**. Concentration (ng-T3 eq/L) of the 13 compounds in site2–site4.	**Page S13**
**Table S11.** Fraction experiment results using the HLB column.	**Pages S14–15**
**Figure S1**. Dose-response and standard curves by Y2H and LC–MS/MS, respectively.	**Page S16**
**Figure S2**. The accurate mass of the main contributor obtained by LC–QTof-MS	**Page S17**

Table S1. Geographic coordinates of the sewage treatment plant (STP) sampling sites in the cities of Tsuchiura (site1), Minato (site2), Sapporo (site3), and Kawasaki (site4). N = north, E = east.

	N	E
**Site 1**	36.084222	140.217028
**Site 2**	35.634809	139.747504
**Site 3**	43.066773	141.414372
**Site 4**	35.596721	139.643595

Table S2. Characteristics of the sewage treatment plant effluent samples collected in the cities of Tsuchiura (site1), Minato (site2), and Kawasaki (site4); Temp. and E.C. indicate the temperature and electrical conductivity, respectively. The site1 samples were collected from June 2020 to May 2021, the site2 ones (site2_Jun. and Sep.) in June 2021 and September 2021, and the site4 one in June 2021.

Site 1	***Jun.***	***Jul.***	***Aug.***	***Sep.***	***Oct.***	***Nov.***
**pH**	6.7	6.9	6.5	6.9	6.2	7.1
**Temp. (°C)**	-	24.9	28.9	28.3	24.2	23.0
**E.C. (ms/cm)**	-	0.44	0.40	0.54	0.50	0.55

Site 1	***Dec.***	***Jan.***	***Feb.***	***Mar.***	***Apr.***	***May***
**pH**	7.3	6.8	6.5	6.6	7.0	6.9
**Temp. (°C)**	21.5	18.4	18.8	21.0	21.1	24.9
**E.C. (ms/cm)**	0.54	0.57	0.54	0.50	0.52	0.70

	Site 2_Jun.	***Site 2_Sep.***	***Site 4***
**pH**	6.7	7.7	8.8
**Temp. (°C)**	26.6	26.6	23.9
**E.C. (ms/cm)**	4.6	5.8	0.61

Table S3. Recovery rates of the 13 compounds by the pretreatment, calculated as the mean of seven replicates. TRIAC=3,3',5-triiodothyroacetic acid; TETRAC=3,3′,5,5′-tetraiodothyroacetic acid; GC-1=sobetirome; T3=3,5,3'-triiodothyronine; T4=3,3',5,5'-tetraiodothyronine; DITPA=3,5-diiodothyropropionic acid; 3-Cl-T3=3-chloro-3′,5,5′-triiodo-L-thyronine; rT3=3,3′,5′-triiodo-L-thyronine; Acetyl T4=N-acetyl L-thyroxine; T1=3-iodo-L-thyronine; TCBPA=tetrachlorobisphenol A; TBBPA=tetrabromobisphenol A.

(μg/L)	1	2	3	4	5	6	7	Average	Recovery rate (%)
**TRIAC**	18	20	18	21	19	18	17	19	**93**
**TETRAC**	14	15	14	16	14	14	13	14	**72**
**GC-1**	26	27	26	28	27	25	24	26	**130**
**T3**	17	16	15	16	15	15	15	16	**78**
**T4**	18	17	17	18	17	17	17	17	**85**
**DITPA**	19	20	19	21	20	19	18	19	**97**
**3-Cl-T3**	17	16	16	16	16	16	16	16	**80**
**rT3**	27	25	26	27	26	25	25	26	**129**
**Acetyl T4**	19	20	20	21	20	19	18	20	**99**
**T1**	13	13	13	13	13	10	10	12	**60**
**TCBPA**	22	24	22	23	23	23	22	23	**114**
**TBBPA**	23	25	23	25	24	26	24	24	**122**
**Triclabendazole**	18	20	18	19	19	17	17	18	**91**

Table S4. Multiple reaction monitoring (MRM) method of the 13 compounds using LC-MS/MS (Triple Quad 5500+).

	Precursor ion	Product ion	Collision energy
TRIAC	576.6	126.7	-40
TETRAC	702.5	126.7	-48
GC-1	327.0	269.1	-32
T3	649.6	126.6	-62
T4	775.5	126.6	-114
DITPA	508.9	126.8	-56
3-Cl-T3	684.5	126.8	-144
rT3	649.6	126.6	-128
Acetyl T4	817.5	126.6	-156
T1	397.9	126.8	-42
TCBPA	364.9	313.5	-36
TBBPA	542.6	78.5	-110
Triclabendazole	358.8	196.9	-50

Table S5. Instrumental detection limits (ag) of the liquid chromatography–tandem mass spectrometry system for the 13 compounds investigated. All the values are represented as the absolute amount in the injection volume (6 µL) and were calculated as the mean of seven replicates.

**TRIAC**	**TETRAC**	**GC-1**	**T3**	**T4**	**DITPA**	**(ag)**
12	17	10	14	17	9.3	
**3-Cl-T3**	**rT3**	**Acetyl T4**	**T1**	**TCBPA**	**TBBPA**	**Triclabendazole**
19	30	31	8.6	18	17	3.4

Table S6. Concentration (ng/L), measured via liquid chromatography–tandem mass spectrometry (LC-MS/MS), of the 13 target compounds in the samples collected from the Tsuchiura sewage treatment plant effluents from June 2020 to May 2021 (site1). All the values were calculated as the mean of three replicates. N.D. means below the limit of quantification (LOQ), whose values for the different compounds were as follows: TRIAC = 0.015 ng/L; TETRAC = 0.015 ng/L; GC-1 = 0.015 ng/L; T3 = 0.015 ng/L; T4 = 0.015 ng/L; DITPA = 0.010 ng/L; 3-Cl-T3 = 0.025 ng/L; rT3 = 0.020 ng/L; acetyl T4 = 0.020 ng/L; T1 = 0.015 ng/L; TCBPA = 0.010 ng/L; TBBPA = 0.020 ng/L, triclabendazole = 0.0050 ng/L.

(ng/L)	*Jun.*	*Jul.*	*Aug.*	*Sep.*	*Oct.*	*Nov.*
TRIAC	0.46	0.86	1.2	0.88	0.75	1.0
TETRAC	*N.D.*	*N.D.*	*N.D.*	*N.D.*	*N.D.*	*N.D.*
GC-1	*N.D.*	*N.D.*	0.0026	*N.D.*	*N.D.*	*N.D.*
T4	*N.D.*	*N.D.*	*N.D.*	*N.D.*	*N.D.*	*N.D.*
T3	0.0051	0.031	0.037	0.013	0.028	*N.D.*
DITPA	*N.D.*	*N.D.*	*N.D.*	*N.D.*	*N.D.*	*N.D.*
3-Cl-T3	*N.D.*	0.050	*N.D.*	*N.D.*	*N.D.*	*N.D.*
rT3	*N.D.*	0.031	0.018	0.030	0.014	*N.D.*
Acetyl T4	*N.D.*	*N.D.*	*N.D.*	*N.D.*	*N.D.*	*N.D.*
T1	0.39	0.50	0.093	0.00024	0.047	0.027
TCBPA	0.079	0.11	0.19	0.14	0.069	0.089
TBBPA	0.052	0.076	0.15	0.11	0.055	0.14
Triclabendazole	0.0016	0.095	0.073	0.0030	0.054	*N.D.*

	Dec.	*Jan.*	*Feb.*	*Mar.*	*Apr.*	*May*
TRIAC	0.75	0.71	0.63	0.72	0.59	0.51
TETRAC	*N.D.*	*N.D.*	*N.D.*	*N.D.*	*N.D.*	*N.D.*
GC-1	*N.D.*	*N.D.*	*N.D.*	*N.D.*	*N.D.*	*N.D.*
T4	*N.D.*	0.019	*N.D.*	*N.D.*	*N.D.*	0.050
T3	*N.D.*	0.018	0.018	*N.D.*	*N.D.*	0.24
DITPA	*N.D.*	*N.D.*	*N.D.*	*N.D.*	*N.D.*	*N.D.*
3-Cl-T3	0.18	0.065	*N.D.*	*N.D.*	0.21	*N.D.*
rT3	0.017	0.037	0.038	*N.D.*	0.032	*N.D.*
Acetyl T4	*N.D.*	*N.D.*	*N.D.*	*N.D.*	*N.D.*	*N.D.*
T1	0.29	*N.D.*	0.23	0.16	0.25	*N.D.*
TCBPA	0.059	0.074	0.10	*N.D.*	0.10	0.030
TBBPA	0.096	0.086	0.052	0.033	0.032	0.032
Triclabendazole	*N.D.*	*N.D.*	*N.D.*	0.0078	0.0058	*N.D.*

Table S7. Concentration (ng/L), measured via LC-MS/MS, of the 13 target compounds in the samples collected from Minato (site2) and Sapporo (site3_EF) sewage treatment plant effluents. The site2 samples were collected in June 2021 and September 2021; the site3 sample was collected in June 2021; the reference water was collected in Kawasaki city (site4) in June 2021. All the values were calculated as the mean of three replicates. N.D. means below the limit of quantification (LOQ), whose values for the different compounds were as follows: TRIAC = 0.015 ng/L; TETRAC = 0.015 ng/L; GC-1 = 0.015 ng/L; T3 = 0.015 ng/L; T4 = 0.015 ng/L; DITPA = 0.010 ng/L; 3-Cl-T3 = 0.025 ng/L; rT3 = 0.020 ng/L; acetyl T4 = 0.020 ng/L; T1 = 0.015 ng/L; TCBPA = 0.010 ng/L; TBBPA = 0.020 ng/L, triclabendazole = 0.0050 ng/L.

(ng/L)	***Site 2_Jun.***	***Site 2_Sep.***	***Site 3_EF***	***Site 4***
**TRIAC**	**4.2**	**2.6**	**0.30**	*N.D.*
**TETRAC**	**0.26**	**0.43**	*N.D.*	*N.D.*
**GC-1**	*N.D.*	*N.D.*	**0.33**	*N.D.*
**T3**	*N.D.*	**0.38**	*N.D.*	**0.067**
**T4**	**0.11**	**0.41**	**0.05**	**0.20**
**DITPA**	*N.D.*	*N.D.*	*N.D.*	*N.D.*
**3-Cl-T3**	*N.D.*	**0.49**	*N.D.*	**24**
**rT3**	*N.D.*	**0.45**	*N.D.*	**0.11**
**Acetyl T4**	*N.D.*	*N.D.*	*N.D.*	*N.D.*
**T1**	*N.D.*	**0.17**	*N.D.*	*N.D.*
**TCBPA**	**0.11**	**0.075**	**0.022**	**1.0**
**TBBPA**	**0.14**	**0.092**	**0.12**	**0.89**
**Triclabendazole**	*N.D.*	*N.D.*	*N.D.*	*N.D.*

Table S8. Human thyroid hormone receptor (hTR) agonist activity in the sewage treatment plant (STP) effluent samples, measured by a yeast two-hybrid assay. The STP samples were collected in the cities of Tsuchiura (site1), Minato (site2), and Sapporo (site3); the reference water samples were collected in Kawasaki city (site4). The site1 samples were collected from June 2020 to May 2021. The site2 ones were collected in June 2021 and September 2021. The site3 and site4 ones were collected in June 2021. N.D. means not detected. All the values are expressed as the T3 equivalent concentration (ng-T3 eq/L).

Site 1_Jun.	***Site 1_Jul.***	***Site 1_Aug.***	***Site 1_Sep.***	***Site 1_Oct.***	***Site 1_Nov.***
154	202	252	301	125	214
Site 1_Dec.	***Site 1_Jan.***	***Site 1_Feb.***	***Site 1_Mar.***	***Site 1_Apr.***	***Site 1_May***
103	157	111	108	114	133
Site 2_Jun.	***Site 2_Sep.***	***Site 3_EF***	***Site 4***	(ng-T3 eq. /L)	
721	533	65	*N.D.*		

Table S9. Comparison with concentration expressed as equivalent to T3 (ng-T3 eq/L) of the 13 target compounds and the overall hTR-agonist activity (ng-T3 eq/L) in the samples collected from the Tsuchiura STP effluents from June 2020 to May 2021. All values were calculated as the mean of three replicates. N.D. means below the limit of quantification, whose values for the different compounds were as follows (ng-T3 eq. /L); TRIAC = 3.1, TETRAC = 1.8, GC-1 = 0.50, T3 = 0.015, T4 = 0.015, DITPA = 5.7×10^{-3}, 3-Cl-T3 = 8.9×10^{-3}, rT3 = 4.9×10^{-3}, Acetyl T4 = 1.4×10^{-3}, T1 = 1.9×10^{-4}, TCBPA = 9.6×10^{-5}, TBBPA = 6.4×10^{-5}, Triclabendazole = 3.7×10^{-6}. Y2H is hTR-agonist activity by a yeast two-hybrid assay. TCRs is total contribution rates.

(ng-T3 eq. /L)	*Jun.*	*Jul.*	*Aug.*	*Sep.*	*Oct.*	*Nov.*
**TRIAC**	**93**	**177**	**256**	**179**	**154**	**205**
**TETRAC**	*N.D.*	*N.D.*	*N.D.*	*N.D.*	*N.D.*	*N.D.*
**GC-1**	*N.D.*	*N.D.*	**8.8×10^{-2}**	*N.D.*	*N.D.*	*N.D.*
**T3**	**5.1×10^{-3}**	**3.1×10^{-2}**	**3.7×10^{-2}**	**1.3×10^{-2}**	**2.8×10^{-2}**	*N.D.*
**T4**	*N.D.*	*N.D.*	*N.D.*	*N.D.*	*N.D.*	*N.D.*
**DITPA**	*N.D.*	*N.D.*	*N.D.*	*N.D.*	*N.D.*	*N.D.*
**3-Cl-T3**	*N.D.*	**1.8×10^{-2}**	*N.D.*	*N.D.*	*N.D.*	*N.D.*
**rT3**	*N.D.*	**7.5×10^{-3}**	**4.4×10^{-3}**	**7.2×10^{-3}**	**3.3×10^{-3}**	*N.D.*
**Acetyl T4**	*N.D.*	*N.D.*	*N.D.*	*N.D.*	*N.D.*	*N.D.*
**T1**	**4.9×10^{-3}**	**6.3×10^{-3}**	**1.2×10^{-3}**	**3.1×10^{-6}**	**5.9×10^{-4}**	**3.4×10^{-4}**
**TCBPA**	**7.6×10^{-4}**	**1.0×10^{-3}**	**1.9×10^{-3}**	**1.3×10^{-3}**	**6.6×10^{-4}**	**8.5×10^{-4}**
**TBBPA**	**1.7×10^{-4}**	**2.4×10^{-4}**	**4.9×10^{-4}**	**3.6×10^{-4}**	**1.8×10^{-4}**	**4.5×10^{-4}**
**Triclabendazole**	**1.2×10^{-6}**	**7.1×10^{-5}**	**5.5×10^{-5}**	**2.2×10^{-6}**	**4.0×10^{-5}**	*N.D.*
**Y2H (ng-T3 eq. /L)**	**154**	**202**	**252**	**301**	**125**	**214**
**TCRs (%)**	**61**	**88**	**102**	**60**	**123**	**96**

(ng-T3 eq. /L)	***Dec.***	***Jan.***	***Feb.***	***Mar.***	***Apr.***	***May***
**TRIAC**	**153**	**145**	**129**	**147**	**121**	**105**
**TETRAC**	*N.D.*	*N.D.*	*N.D.*	*N.D.*	*N.D.*	*N.D.*
**GC-1**	*N.D.*	*N.D.*	*N.D.*	*N.D.*	*N.D.*	*N.D.*
**T3**	*N.D.*	1.8×10^{-2}	1.8×10^{-2}	*N.D.*	*N.D.*	**0.2**
**T4**	*N.D.*	1.9×10^{-2}	*N.D.*	*N.D.*	*N.D.*	5.0×10^{-2}
**DITPA**	*N.D.*	*N.D.*	*N.D.*	*N.D.*	*N.D.*	*N.D.*
**3-Cl-T3**	6.2×10^{-2}	2.3×10^{-2}	*N.D.*	*N.D.*	7.3×10^{-2}	*N.D.*
**rT3**	4.0×10^{-3}	9.1×10^{-3}	9.3×10^{-3}	*N.D.*	7.8×10^{-3}	*N.D.*
**Acetyl T4**	*N.D.*	*N.D.*	*N.D.*	*N.D.*	*N.D.*	*N.D.*
**T1**	3.6×10^{-3}	*N.D.*	2.9×10^{-3}	2.0×10^{-3}	3.2×10^{-3}	*N.D.*
**TCBPA**	5.7×10^{-4}	7.1×10^{-4}	9.6×10^{-4}	*N.D.*	9.8×10^{-4}	2.9×10^{-4}
**TBBPA**	3.1×10^{-4}	2.8×10^{-4}	1.7×10^{-4}	1.0×10^{-4}	1.0×10^{-4}	1.0×10^{-4}
**Triclabendazole**	*N.D.*	*N.D.*	*N.D.*	5.8×10^{-6}	4.3×10^{-6}	*N.D.*
**Y2H (ng-T3 eq. /L)**	**103**	**157**	**111**	**108**	**114**	**133**
**TCRs (%)**	**148**	**92**	**116**	**136**	**106**	**79**

Table S10. Comparison with concentration expressed as equivalent to T3 (ng-T3 eq/L) of the 13 target compounds and the overall hTR-agonist activity (ng-T3 eq/L) in the samples collected from the site2, site3, and site4. The site2 samples are STP effluents collected in June 2021 and September 2021; the site3 sample is a STP effluent collected in June 2021; the site4 sample is a reference water collected June 2021. All the values were calculated as the mean of three replicates. N.D. means below the limit of quantification, whose values for the different compounds were as follows (ng-T3 eq. /L); TRIAC = 3.1, TETRAC = 1.8, GC-1 = 0.50, T3 = 0.015, T4 = 0.015, DITPA= 5.7×10^{-3}, 3-Cl-T3 = 8.9×10^{-3}, rT3 = 4.9×10^{-3}, Acetyl T4 = 1.4×10^{-3}, T1 = 1.9×10^{-4}, TCBPA = 9.6×10^{-5}, TBBPA = 6.4×10^{-5}, Triclabendazole = 3.7×10^{-6}. Y2H is hTR-agonist activity by a yeast two-hybrid assay. TCRs is total contribution rates.

(ng-T3 eq. /L)	*Site 2_Jun.*	*Site 2_Sep.*	*Site 3_EF*	*Site 4*
**TRIAC**	**860**	**533**	**61**	*N.D.*
**TETRAC**	**32**	**52**	*N.D.*	*N.D.*
**GC-1**	*N.D.*	*N.D.*	**11**	*N.D.*
**T3**	*N.D.*	**0.38**	*N.D.*	**6.7×10^{-2}**
**T4**	**0.11**	**0.41**	**5.4×10^{-2}**	**0.2**
**DITPA**	*N.D.*	*N.D.*	*N.D.*	*N.D.*
**3-Cl-T3**	*N.D.*	**0.17**	*N.D.*	**8.5**
**rT3**	*N.D.*	**0.11**	*N.D.*	**2.7×10^{-2}**
**Acetyl T4**	*N.D.*	*N.D.*	*N.D.*	*N.D.*
**T1**	*N.D.*	**2.1×10^{-3}**	*N.D.*	*N.D.*
**TCBPA**	**1.1×10^{-3}**	**7.2×10^{-4}**	**2.1×10^{-4}**	**9.6×10^{-3}**
**TBBPA**	**4.5×10^{-4}**	**3.0×10^{-4}**	**3.8×10^{-4}**	**2.9×10^{-3}**
**Triclabendazole**	*N.D.*	*N.D.*	*N.D.*	*N.D.*
**Y2H (ng-T3 eq. /L)**	**721**	**533**	**65**	-
**TCRs (%)**	**124**	**110**	**111**	**-**

Table S11. Behavior evaluation of TRIAC and hTR-agonist activity by fractionation using a HLB column (ng-T3 eq/L). All values were calculated as the mean of three replicates. N.D. means below the limit of quantification, whose values for the different compounds were as follows (ng-T3 eq. /L); TRIAC = 3.1, TETRAC = 1.8, GC-1 = 0.50, T3 = 0.015, T4 = 0.015, DITPA = 5.7×10^{-3}, 3-Cl-T3 = 8.9×10^{-3}, rT3 = 4.9×10^{-3}, Acetyl T4 = 1.4×10^{-3}, T1 = 1.9×10^{-4}, TCBPA = 9.6×10^{-5}, TBBPA = 6.4×10^{-5}, Triclabendazole = 3.7×10^{-6}. Y2H is hTR-agonist activity by a yeast two-hybrid assay, and N.A. means no activity. TCRs is total contribution rates.

(ng-T3 eq. /L)	MeOH_10%	MeOH_20%	MeOH_30%	MeOH_40%	MeOH_50%	MeOH_60%
**TRIAC**	*N.D.*	*N.D.*	*N.D.*	*N.D.*	*N.D.*	*N.D.*
**TETRAC**	*N.D.*	*N.D.*	*N.D.*	*N.D.*	*N.D.*	*N.D.*
**GC-1**	*N.D.*	*N.D.*	*N.D.*	*N.D.*	*N.D.*	*N.D.*
**T4**	*N.D.*	*N.D.*	*N.D.*	*N.D.*	*N.D.*	*N.D.*
**T3**	*N.D.*	*N.D.*	*N.D.*	*N.D.*	*N.D.*	*N.D.*
**DITPA**	*N.D.*	*N.D.*	*N.D.*	*N.D.*	*N.D.*	*N.D.*
**3-Cl-T3**	*N.D.*	*N.D.*	*N.D.*	*N.D.*	*N.D.*	*N.D.*
**rT3**	*N.D.*	*N.D.*	*N.D.*	*N.D.*	*N.D.*	*N.D.*
**Acetyl T4**	*N.D.*	*N.D.*	*N.D.*	*N.D.*	*N.D.*	*N.D.*
**T1**	*N.D.*	*N.D.*	*N.D.*	*N.D.*	*N.D.*	*N.D.*
**TCBPA**	*N.D.*	*N.D.*	*N.D.*	*N.D.*	*N.D.*	*N.D.*
**TBBPA**	*N.D.*	*N.D.*	*N.D.*	*N.D.*	*N.D.*	*N.D.*
**Triclabendazole**	*N.D.*	*N.D.*	*N.D.*	*N.D.*	*N.D.*	*N.D.*
**Y2H (ng-T3 eq. /L)**	*N.A.*	*N.A.*	*N.A.*	*N.A.*	*N.A.*	*N.A.*
**TCRs (%)**	-	-	-	-	-	-

(ng-T3 eq. /L)	MeOH_70%	MeOH_80%	MeOH_90%	MeOH_100%	No fraction
**TRIAC**	*N.D.*	*N.D.*	**98**	**39**	**124**
**TETRAC**	*N.D.*	*N.D.*	*N.D.*	**0.46**	*N.D.*
**GC-1**	*N.D.*	*N.D.*	*N.D.*	*N.D.*	*N.D.*
**T4**	*N.D.*	*N.D.*	*N.D.*	*N.D.*	*N.D.*
**T3**	*N.D.*	*N.D.*	*N.D.*	*N.D.*	*N.D.*
**DITPA**	*N.D.*	*N.D.*	*N.D.*	*N.D.*	*N.D.*
**3-Cl-T3**	*N.D.*	*N.D.*	*N.D.*	*N.D.*	*N.D.*
**rT3**	*N.D.*	*N.D.*	*N.D.*	*N.D.*	*N.D.*
**Acetyl T4**	*N.D.*	*N.D.*	*N.D.*	*N.D.*	*N.D.*
**T1**	*N.D.*	*N.D.*	*N.D.*	*N.D.*	*N.D.*
**TCBPA**	*N.D.*	*N.D.*	3.4×10^{-4}	2.9×10^{-4}	7.1×10^{-4}
**TBBPA**	*N.D.*	*N.D.*	*N.D.*	8.0×10^{-5}	1.3×10^{-4}
**Triclabendazole**	*N.D.*	*N.D.*	*N.D.*	*N.D.*	*N.D.*
**Y2H (ng-T3 eq. /L)**	*N.A.*	*N.A.*	**260**	**55**	**341**
**TCRs (%)**	-	-	**38**	**71**	**36**

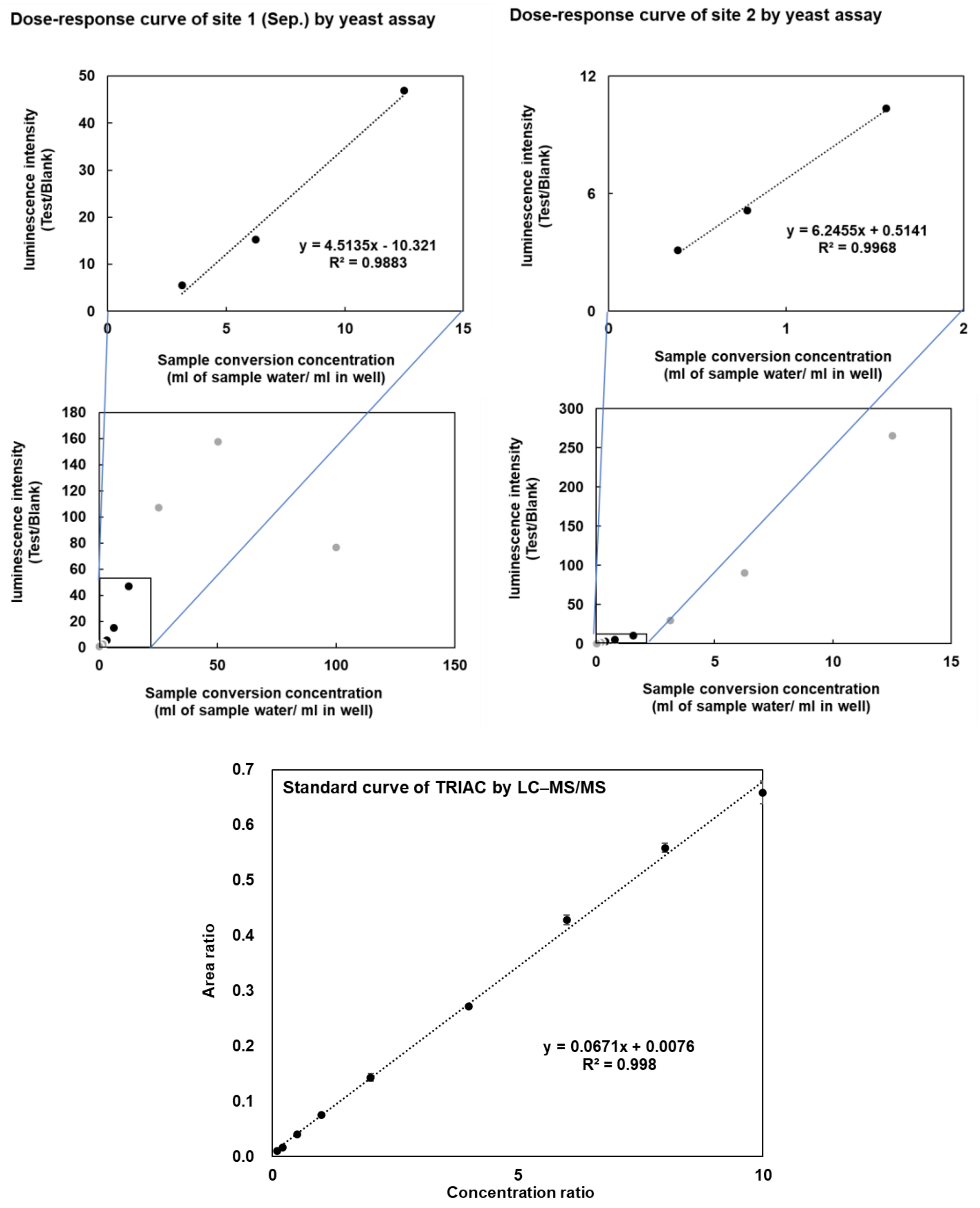

Figure S1. Dose-response curves by the yeast assay and standard curve by LC-MS/MS.
An error bar represents standard errors (n = 3), and some standard errors were smaller than the symbols used to represent the means.

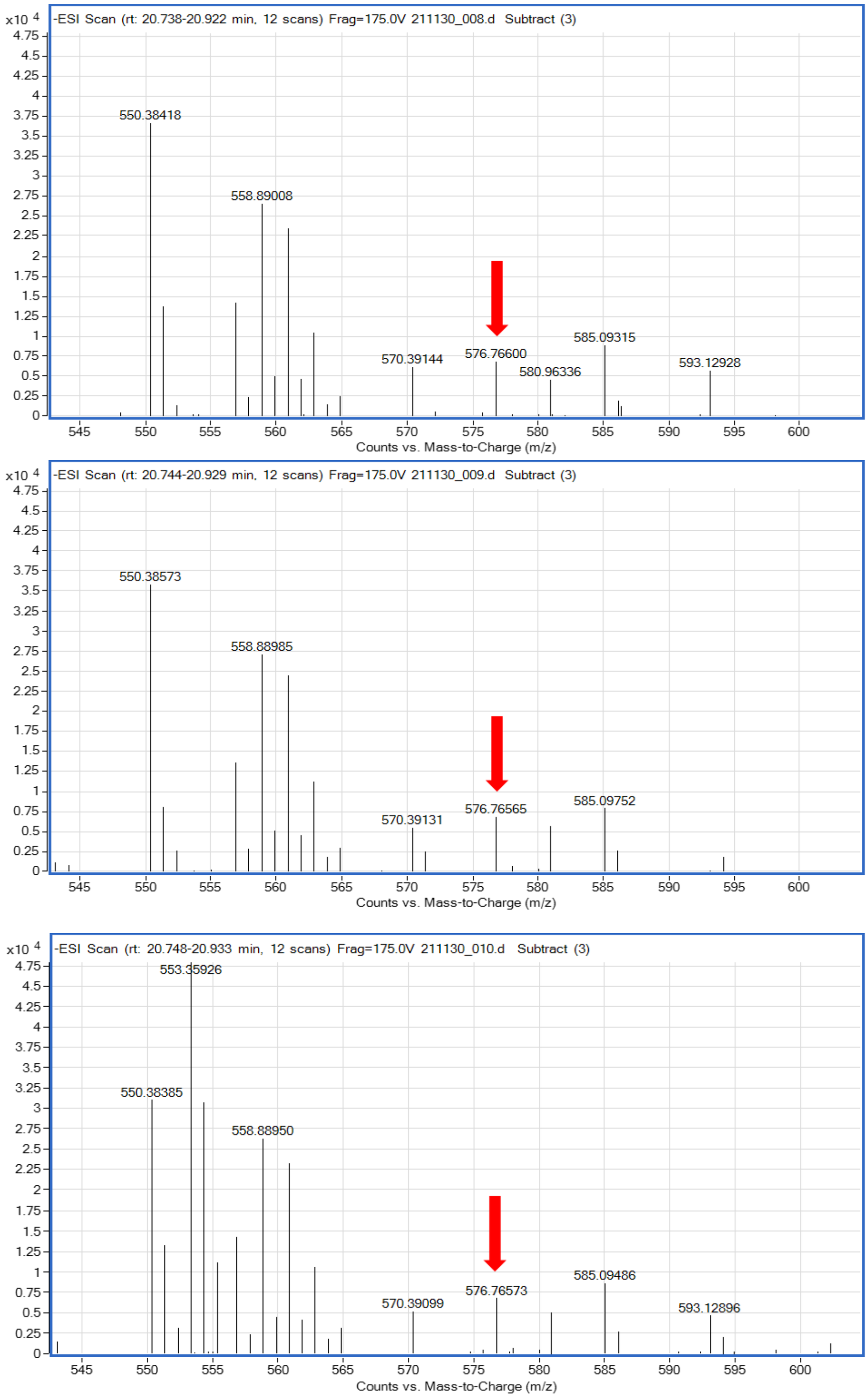

Figure S2. Mass spectra measured via liquid chromatography-quadrupole time-of-flight mass spectrometry, including the accurate masses of compounds like TRIAC (indicated by the red arrows), of the STP effluent samples collected in Minato city (site2_Sep).
